# Protection against SHIV-KB9 Infection by Combining rDNA and rFPV Vaccines Based on HIV Multiepitope and p24 Protein in Chinese Rhesus Macaques

**DOI:** 10.1155/2012/958404

**Published:** 2012-02-26

**Authors:** Chang Li, Zhenwei Shen, Xiao Li, Jieying Bai, Lin Zeng, Mingyao Tian, Ying Jin Song, Ming Ye, Shouwen Du, Dayong Ren, Cunxia Liu, Na Zhu, Dandan Sun, Yi Li, Ningyi Jin

**Affiliations:** ^1^Institute of Military Veterinary Medicine, Academy of Military Medical Sciences, LiuYingXi Road No. 666, Changchun 130122, China; ^2^Laboratory Animal Center, Academy of Military Medical Sciences, Dongda Street No. 20, Beijing 100071, China; ^3^School of Agriculture and Biological Engineering, Tianjin University, Weijin Road No. 92, Tianjin 300072, China

## Abstract

Developing an effective vaccine against HIV infection remains an urgent goal. We used a DNA prime/fowlpox virus boost regimen to immunize Chinese rhesus macaques. The animals were challenged intramuscularly with pathogenic molecularly cloned SHIV-KB9. Immunogenicity and protective efficacy of vaccines were investigated by measuring IFN-*γ* levels, monitoring HIV-specific binding antibodies, examining viral load, and analyzing CD4/CD8 ratio. Results show that, upon challenge, the vaccine group can induce a strong immune response in the body, represented by increased expression of IFN-*γ*, slow and steady elevated antibody production, reduced peak value of acute viral load, and increase in the average CD4/CD8 ratio. The current research suggests that rapid reaction speed, appropriate response strength, and long-lasting immune response time may be key protection factors for AIDS vaccine. The present study contributes significantly to AIDS vaccine and preclinical research.

## 1. Introduction

Human immunodeficiency virus type 1 (HIV-1) is the aetiological agent of acquired immune deficiency syndrome (AIDS). Since the first case of HIV-1 infection was reported in Los Angeles in 1981 [1], more than 68 million people have been infected with HIV worldwide. Nearly 25 million people have died, and approximately 33.3 million people are suffering from HIV globally at the end of 2009 according to the 2010 global report published by the Joint United Nations Programme on HIV/AIDS [2]. AIDS/HIV remains the deadliest crisis and the greatest social, economic, and public health challenge in modern times because of the absence of effective prophylaxis or therapy methods [3–9].

Undoubtedly, the best and most economical solution to eradicate or control the spread of HIV-1 is to develop safe and effective vaccine. Although considerable efforts have been devoted toward this goal, the success of available vaccines has not been demonstrated [3, 4, 10, 11]. Previous studies have determined that the HIV-specific CD8^+^ cytotoxic T-cell responses play key roles in controlling viral replication, which could reduce viral loads and postpone disease progression in individuals who are infected with HIV-1 [12–16]. Immunization with polyvalent antigens may likely stimulate more effective immunity than a single antigen as described in an earlier study [12, 17]. Thus, combining multi-CTL epitopes derived from different genes of HIV-1 to construct a chimeric antigen may be a better strategy to develop new vaccines. Previously, we designed and constructed a new immunogen, which includes 29 multiepitopes and the p24 protein of HIV-1 as carrier molecule. The selected epitopes covered the most dominant epitopes derived from structural, regulatory, and accessory proteins of HIV, such as Gag, Env, Pol, RT, IN, Vpr, Tat, and Nef; HLA-DR epitope, ER signal peptide, and Kozak sequence were considered as well. The antigenicity and immunogenicity were evaluated in vitro and in vivo [18–20].

To further confirm the immunogenicity and protective effect of this vaccine, we immunized Chinese rhesus macaques with a DNA prime/fowlpox virus boost regimen. These animals were challenged intravenously with pathogenic molecularly cloned SHIV-KB9. The monkeys were monitored by measuring their IFN-*γ* levels, HIV-specific binding antibodies, viral load, and CD4/CD8 ratio and by analyzing the immunogenicity and the protective effect of vaccine to facilitate clinical trials. 

## 2. Materials and Methods

### 2.1. Animals

Chinese-origin rhesus macaques (Ch Rh), 3–5 years of age, both female and male, and with no signs of clinical diseases, were provided by the Laboratory Animal Center, Academy of Military Medical Sciences. Sixteen Chinese rhesus macaques from Guangxi, aged 3–5 years, weighing 3–5 kg, and without simian immunodeficiency virus (SIV), monkey T lymphocytes of I virus (STLV), monkey ART D-type virus (SRV/D), or B virus infection, were bred and provided by the experimental Animal Center of Military Medical Sciences. The present research project was approved by the relevant ethics review committee. Animal husbandry and sample collection were in accordance with relevant biosecurity requirements.

### 2.2. Vaccines

**Figure 1:**
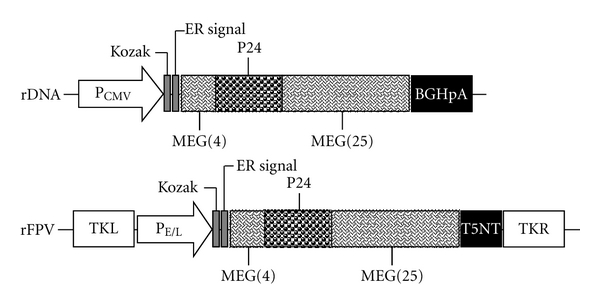
Schematic representation of the rDNA and rFPV vaccine constructs. The functional elements of the expression vector are the following. P_CMV_: human cytomegalovirus (CMV) immediate-early promoter/enhancer; Kozak: a Kozak translation-initiation sequence and an initiation codon (ATG) for proper initiation of translation; ER signal: endoplasmic reticulum signal peptide; MEG(4): multi-epitope gene (including 4 epitopes); P24: HIV-1 capsid protein; MEG(25): multi-epitope gene (including 25 epitopes); BGHpA: Bovine growth hormone (BGH) polyadenylation signal; TKL: the left recombinant fowlpox virus; P_E/L_: early and late promoter of fowlpox virus; T5NT: terminal signal of fowlpox virus; TKR: the right recombinant fowlpox virus.

The vaccines used in the current study are recombinant DNA vaccine rDNA/pVMp24 and recombinant fowlpox virus rFPV/Mp24. Both are epitope-based vaccines containing the same immunogens, which includes a Kozak translation initiation sequence, ER signal peptide, 29 HIV dominant epitopes (24 CTL or CD8 T-cell epitopes and 5 B-cell epitopes), and HIV-1 p24 protein. The immunogens were provided by professor Ningyi Jin of the Institute of Military Veterinary Medicine, Academy of Military Medical Sciences. The schematic representation of the rDNA and rFPV vaccine constructs is shown in Figure 1.

### 2.3. Immunization and Challenge Experiments

The Chinese rhesus macaques were randomly divided into 2 groups (4 macaques per group). Each group was primed intramuscularly (i.m.) with rDNA/pVMp24 (500 *μ*g/per animal) vaccine and empty vector pVAX1 control at weeks 0, 4, and 10 and subsequently boosted with 10^9^ plaque-forming unit (PFU) rFPV/Mp24 vaccine and wild-type FPV at week 18. At week 28, the macaques were challenged intravenously with 20 MID_50_ of SHIV-KB9 provided by professor Yiming Shao of the Chinese Center for Disease Control and Prevention.

**Figure 2:**
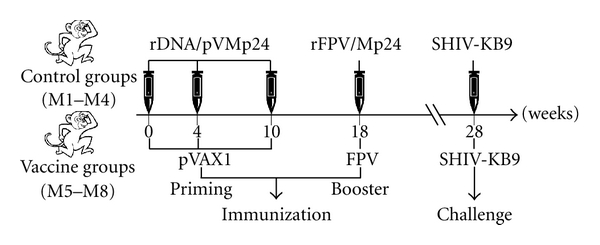
Immunization and challenge schedule. rDNA: recombinant DNA; rFPV: recombinant FPV.

The immunization schedule and routes of administration are outlined in Figure 2.

### 2.4. Sample Collection and Processing

In the presence of EDTA anticoagulant, hind-limb venous blood was collected at 7, 13, 21, 28, and 35 d postinfection. The samples were sent to the laboratory within 6 h. Part of the unclotted blood was processed for blood routine examination and flow analysis. The remaining samples were centrifuged at 1700 rpm and kept at room temperature for 10 min. Plasma was analyzed for virus RNA load and antibodies. PBMC was separated from the blood cell for enzyme-linked immunospot (ELISPOT) and other analyses. Plasma was stored at −20°C, and the PBMC was frozen in liquid nitrogen.

### 2.5. IFN-*γ* ELISPOT Detection

ELISPOT assays were conducted to evaluate the gamma interferon-(IFN-*γ*-)secreting cells. PBMCs were isolated by Ficoll gradient centrifugation as previously described [21]. ELISPOT responses were detected using the monkey IFN-*γ* ELISPOT kit (U-CyTech Biosciences, Utrecht, the Netherlands) according to the instructions of the manufacturer. Each sample was stimulated in triplicate by adding a single pool of p24 peptides (15-mer HIV-1 consensus p24 peptides with an 11-amino-acid overlap, synthesized by HD Biosciences Co., Ltd., Shanghai, China) with a final concentration of 4 *μ*g/mL for each peptide. PMA (50 ng/mL) and ionomycin (1 *μ*g/mL) were used as positive controls, whereas RPMI 1640 medium was used as negative control. The results were indicated as spot-forming cells (SFC)/million PBMC. A positive response was defined as 4 times ELISPOT points higher than the negative control points and greater than 50 SFC/10^6^ cells at each time point.

### 2.6. Detection of HIV-1-Specific Binding Antibodies in the Serum

Sequence alignment indicates that HIV-1 p24 and SHIV p24 proteins have strong homology. Thus, the antibodies induced by the SHIV virus have a strong cross-immune response to the HIV antigen. In the present study, the HIV-1-specific antibody responses were measured by the third-generation total HIV-binding antibody diagnostic kit (Vironostika HIV Uni-Form II Plus O, BioMérieux Corporate, France). The experiment was conducted by employing the enzyme-linked immunosorbent assay according to the protocols of the manufacturer and the literature.

### 2.7. Measurement of Plasma Viral RNA Load

The plasma viral RNA was extracted by QIAGEN Viral RNA minikit (QIAGEN company) and analyzed by real-time PCR using TaqMan EZ RT-PCR Core Reagents kit (ABI Company) and ABI Prism 7700 apparatus. SHIV viral RNA in the samples was quantified by the standard curve derived from RNA standards.

### 2.8. CD3, CD4, and CD8 Lymphocyte Subset Analysis

We used three nonhuman primate antibodies, FITC-CD3, PerCP-CD4, and PE-CD8 (BD Pharmingen Inc.) in the flow analysis. Each flow tube contained 5 *μ*L of each antibody and 100 *μ*L of whole blood. Bland control and CD3-, CD4-, and CD8-stained controls were set up. The contents were mixed evenly by shaking and incubated under darkness at room temperature for 20 min. During shaking, 1 mL 1 x PBS dissolved in 500 *μ*L hemolysin was added to lyse the red blood cells. The mixture was kept for 10 min until the liquid became translucent. Subsequently, the mixture was centrifuged at 1500 rpm for 5 min. The supernatant was discarded and the cells were scattered by vibration. Formaldehyde fixative (300 *μ*L) was added and the samples were analyzed by flow cytometry (BD FACSCalibur).

### 2.9. Statistical Analysis

Differences between the groups were analyzed by Student's *t*-test. The results were expressed as mean ± SD. *P* value < 0.05 was considered significant. 

## 3. Results

### 3.1. ELISPOT Test of IFN-*γ*

**Figure 3:**
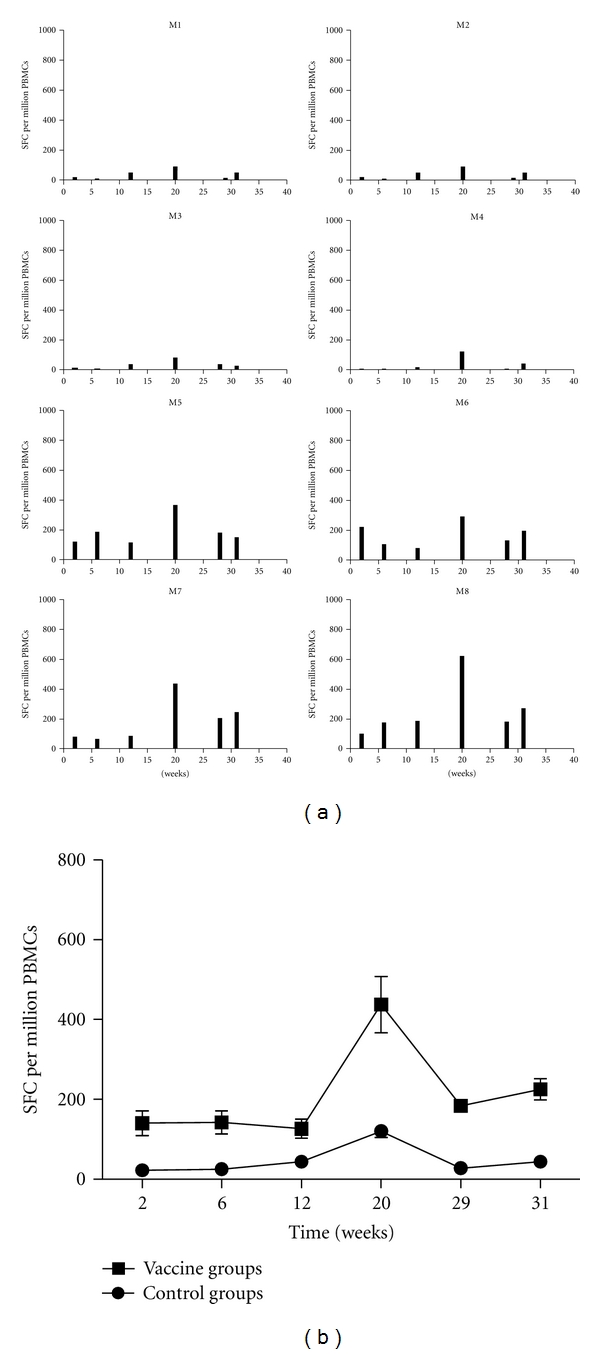
Number of IFN-*γ*-secreting PBMC was detected by ELISPOT after stimulation with p24 peptide pools at various times after immunization and challenge. (a) Histogram bars represent the average number of spots per million cells detected in duplicate cultures for each animal, after subtraction of the average number of spots found in duplicate control cultures of PBMC without stimulation. (b) Dynamics of the IFN-*γ* response was observed throughout the experiment for each group.

ELISPOT method was used to determine the IFN-*γ*-secreting T-cell immune responses stimulated by HIV-1 p24 peptide library. Compared with the control animals, the vaccine group produced strong ELISPOT positive responses after immunization, as shown in Figure 3. Two weeks after rFPV booster immunization (20 w), the ELISPOT response was significantly enhanced, with an average of 437SFC/10^6^ cells. One empty vector also showed weak positive responses, indicating that fowlpox virus vector itself has certain nonspecific T-cell responses.

After the SHIV-KB9 virus attacks, all animals in the immunized group showed different degrees of ELISPOT-positive responses (peak in the range of 115–890 SFC/10^6^ cells). At day 7 postinfection (29 w), a rapid increase in ELISPOT response was detected, and at day 21 (31 w), the ELISPOT response remained at an appropriate response level. These results suggest that the vaccine produced in the present study has good cellular memory immune response.

### 3.2. Measurement of Serum-Specific Binding Antibodies

The antibody analysis results after infection are shown in Table 1. The control group (A) showed weak positive response at day 35 (M1-M2). M3 showed positive response at days 28 and 35, but the antibody titers did not increase. However, antibody titers of all animals in the vaccine group showed slow, steady rise. The antibody production time was significantly earlier (M5, M7, and M8 at day 21) than the other group, indicating that the vaccine induced significant humoral immune memory response.

### 3.3. Measurement of Plasma Viral RNA Load

**Figure 4:**
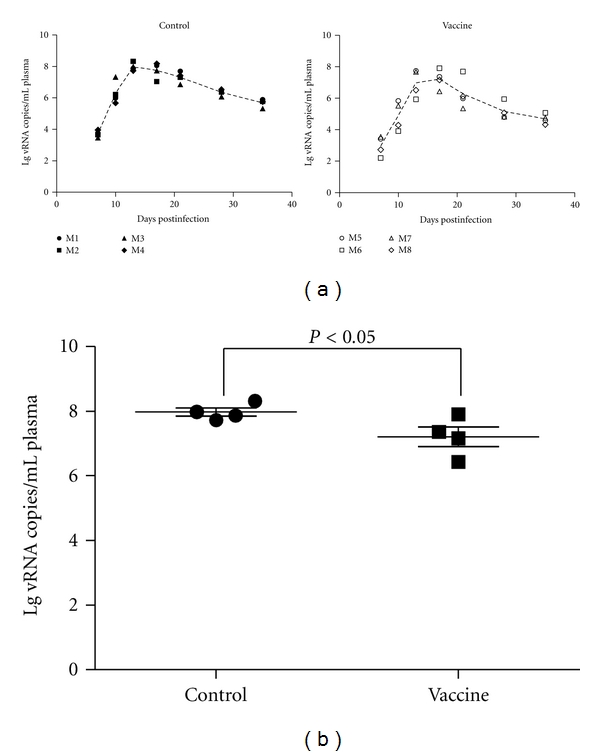
Plasma viral load analysis post-SHIV-KB9 challenge. Viremia was quantified by RT-PCR. (a) Dynamics of viral load for each group. (b) Average value of viral load for each group.

The results of plasma viral RNA load are shown in Figure 4. The peak value of average viral load appeared at day 17 in all the vaccine groups, which was later than that in the control group (13 d). The peak value of the average was significantly lower than that of the negative control group (*P* < 0.05), indicating that the vaccine has certain inhibitory effects on virus replication.

### 3.4. T-Lymphocyte Subset Analysis

**Figure 5:**
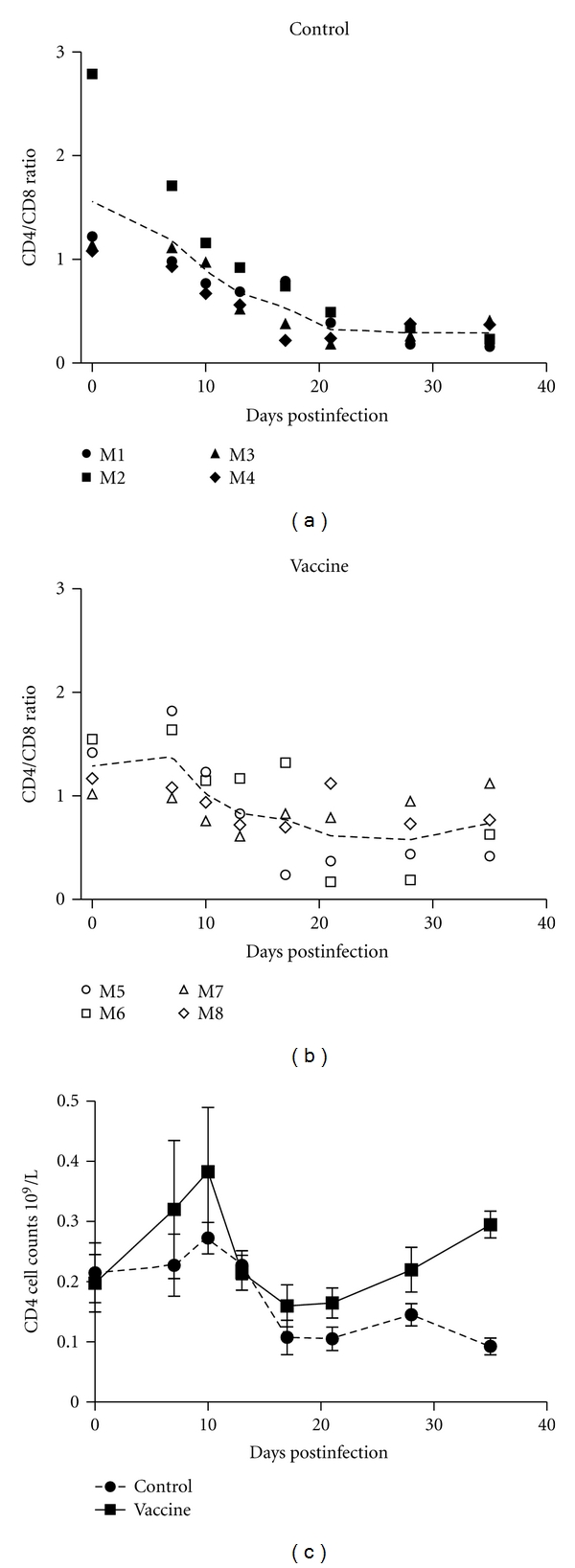
CD4/CD8 ratio analysis and total CD4 counts post-SHIV-KB9 challenge.

Flow analysis of the T-lymphocyte subsets is shown in Figure 5. When the rhesus macaques were infected by the virus, all the animals in the control group exhibited continuous decline in terms of CD4/CD8 ratio, with the inversion phenomenon occurring at day 13. During the entire experimental time, no recovery of CD4/CD8 was detected. However, the overall average ratio of CD4/CD8 in the vaccine group declined at first and subsequently increased, and the average ratio of CD4/CD8 recovered to a relatively higher level at day 35.

## 4. Discussion and Conclusion

SIV/rhesus macaques are the most effective models for the investigation of the mechanisms for HIV pathogenesis and prevention [22, 23]. However, the antigenic differences among SIV, HIV-1, and HIV-2 cause significant limitations to this model [24–26]. In recent years, the use of chimeric simian/human immunodeficiency virus (SHIV) instead of SIV as an infection model has increased [27]. Previously, most HIV vaccine trials in monkeys involved Indian rhesus macaques [28, 29]. The SHIV-KB9/Indian rhesus model is widely used in numerous research institutions and has become a reference model for the evaluation of the immune protective effects of various vaccines [30, 31]. However, due to the shortage of Indian rhesus animal resources, the Chinese rhesus population (comprising approximately 30 million Chinese wild rhesus macaques) has become an important alternative source [32]. Previous studies have suggested that Chinese rhesus macaques (ChR) are better models for AIDS vaccine research [33–35]. The current study made use of the SHIV/Chinese rhesus model to evaluate vaccine immunogenicity and protective efficacy. 

Previous studies have shown that natural viral antigen may contain components with negative effects on protective responses including several immune suppression and immune pathological sequences. These components can interfere with the immune response and block the cell signaling pathway, leading to loss of balance for Th1/Th2 type immune response in the body [4, 36–39]. Consequently, this can result in immune response deviation or defect. Therefore, screening, alteration, or modification of the natural antigen at the level of epitope to remove negative factors on immune response while ensuring response specificity is extremely important for vaccine designing. In addition, vaccines with only single antigen gene have no significant immune effects. Thus, the vaccine design needs to incorporate multiple and different immunogens as well as structural variety to obtain broad virus-specific immune response [17, 40, 41]. Thus, the current research presents a multi-epitope gene based on the advantageous HIV epitope using macromolecular particle protein p24 as carrier molecule. The multi-epitope gene contains HIV-specific T-cell epitope, HIV-specific B-cell epitope, universal Th epitope, B-cell epitope, and one B-cell epitope from tetanus toxin (TT). In addition to the function of structural proteins such as Gag, Env, and Pol, the vaccine also strengthens the important role of nonstructural proteins (vpr, nef, tat, etc.) in immune response and viral replication control. For chronic infections, such as HIV, the cellular immune response, particularly CTL, is of considerable significance to clear virus-infected cells [42–44]. Thus, we emphasized the epitopes of nonstructural proteins of HIV, and these epitopes were selected mainly from the classic, advantageous kind with conservation capacity, broad cross-reactivity, and have been proven in both patients and animal experiments. This will allow focusing on the nonstructural protein CTL of HIV. Considering the new features of domestic and Asian HIV epidemic, the epitope was adjusted based on the recently published HIV subtype at GenBank (Genbank Accession Number AX149898). During the vaccine construction, Kozak rules were considered. Peptide signal sequence, codon preference, and other factors that can increase antigen transcription and translation were targeted, with the goal of achieving an effective candidate vaccine with induction-specific immune response to break the immune resistance to HIV antigens in the host. Essentially, the aims are preventing and controlling HIV infection as well as providing remedy to the disease after vaccination.

“Prime-boost” immunization strategy is a sensational topic in current vaccine study [45]. The first reported use of this strategy was in the immunization of influenza virus [46]. Currently, this immunization strategy is widely used in the research of a variety of pathogen vaccines, especially in AIDS vaccine study [17, 41, 47].

In the study, we used an rDNA/rFPV “prime-boost” coimmunization strategy to immunize the Chinese rhesus macaques. Moreover, we used the SHIV-KB9 infection to analyze the immune protective effect of the vaccine. The results show that, during the primary immunization of rDNA/pVMp24 vaccine, the vaccine only induced a relatively low level of IFN-*γ*-secreting T-cell immune response in the immunized group, whereas after rFPV booster immunization, the immune response significantly increased. This is consistent with the related literature [41, 48]. The primary immunization of DNA vaccines can induce high-affinity T cells, but with low levels of immunity. However, after fowlpox virus booster immunization, the fowlpox enhances the immune effect of primary immunization through proinflammatory immune response of the body.

IFN-*γ*-secreting T-cell immune response analysis performed one week and three weeks after the SHIV-KB9 virus attack shows that when exposed to the virus, all the vaccinated groups quickly activated antigen-specific CD8^+^ T-cell immunity. Rapid increase in ELISPOT response was detected 7 days after infection and was maintained at an appropriate response level at day 21. This indicates that the vaccine effectively extends the memory CD8^+^ T-cell survival and maintains the capability of T-cell immune responses.

With regard to humoral immunity, we focused on the production rate of p24 antibodies, antibody titers, and antibody duration. The M1 and M2 in the negative control group showed weak HIV-1 binding antibody response after 35 days of virus attack. M3 showed positive response at days 28 and 35. However, the antibody titers did not rise. M4 remained negative during the experiment. Three animals in the immunized group (M5, M7, M8) showed positive antibody response at day 21, and the antibody titers exhibited a smooth, steady rise over time, indicating that the immunized group effectively induced the production of specific antibodies.

Viral load is a major parameter which can be used to evaluate whether HIV vaccine can induce immune protection, and the viral load change after the viral attack predicts the progress of the disease. In the present study, the positive response of viral load appeared in the control animals after 7 days of viral attack, and the peak value of average load occurred at day 13. In contrast, the peak value of the average load occurred at day 17 in the immunized group, and the peak value was lower than that in the control group. These results suggest that the vaccine delays the production of virus peak point at the early-infection stage and reduces the viral load at the peak point.

After virus infection, all the rhesus macaques in the negative control group exhibited the inversion phenomenon in CD4/CD8 ratio at day 13, and the ratio continued to decline rapidly. No recovery of CD4/CD8 ratio was observed during the experiment. However, in the immunized animals, the average CD4/CD8 ratio decreased at first, subsequently increased, and finally was restored to a relatively higher level at day 35. This indicates that the vaccine effectively induced T-cell proliferation.

The fowlpox virus expression system in the study is a newly developed poxvirus vector based on vaccinia virus vector [48, 49]. The vector inherits the same advantages of vaccinia virus vectors. Furthermore, the fowlpox virus has a narrow host range and more safe [50, 51]. Except for consideration on vectors, the immunogen design is also a crucial factor for good vaccine. In the present study, we selected and designed an optimal antigen combination containing HIV-1 advantageous epitope by scanning and analyzing the entire HIV-1 genome, searching the authoritative database, and referring to the new features of Asian and domestic HIV epidemic strains. In addition, we utilized p24 protein as a molecular scaffold. Based on the secure plasmid DNA and the new FPV transfer vector, we constructed the AIDS vaccine containing the above-mentioned antigens. The preliminary monkey immune experiments and infection study show that this vaccine can decrease the peak value of viral load at acute phase, delay the peak time, and make the viral load rapidly decline. The results indicated that the vaccine constructed in the current study has certain immune protection effects. And this provides new ideas and methods for AIDS vaccine development. We are currently conducting the safety, quality control, and other preclinical researches for this vaccine so as to move forward to the next phase of the clinical study.

## Figures and Tables

**Table 1:** Whole-virus HIV-specific binding antibody titers after the challenge.

Groups	Animals	Whole-virus HIV-1 specific antibody titers				
		7 days	14 days	21 days	28 days	35 days
Control	M1	0	0	0	0	1
	M2	0	0	0	0	1
	M3	0	0	0	1	1
	M4	0	0	0	0	0
Vaccine	M5	0	0	1	1	3
	M6	0	0	0	1	2
	M7	0	0	1	1	2
	M8	0	0	1	2	3
